# Inhibition of DNA demethylation attenuates experimental necrotizing enterocolitis via suppression of TLR4-mediated inflammation

**DOI:** 10.21203/rs.3.rs-10270484/v1

**Published:** 2026-07-08

**Authors:** Yukihiro Yamaguchi, Vikram Puri, Corey M. Jania, Olivia G. Cassidy, Lauren R. Bolzan, Gergely D. Mozes, Ethan A. Brown, Sofia Domenech, Claire M. Miller, Adelle B. Levi, Ahmad Salah Sami, Tessa-Jonne F. Ropp, Danielle E. Sklar, David G. Peters, Natalia S. Akopyants, Misty Good

**Affiliations:** University of North Carolina School of Medicine; University of North Carolina School of Medicine; University of North Carolina School of Medicine; University of North Carolina School of Medicine; University of North Carolina School of Medicine; University of North Carolina School of Medicine; University of North Carolina School of Medicine; University of North Carolina School of Medicine; University of North Carolina School of Medicine; University of North Carolina School of Medicine; Seattle Children’s Hospital; University of North Carolina School of Medicine; University of North Carolina School of Medicine; University of Pittsburgh; University of North Carolina School of Medicine; University of North Carolina School of Medicine

**Keywords:** necrotizing enterocolitis, TLR4, DNA demethylation, TET enzymes, TET inhibitor

## Abstract

**Background:**

Necrotizing enterocolitis (NEC) is a serious inflammatory bowel disease that primarily affects preterm infants. NEC is characterized by damage to the intestinal epithelium, inflammation, and a high mortality rate. Alterations in gene regulation, particularly DNA methylation, have been linked to the development of NEC in preterm infants. However, the significance of DNA demethylation and its potential as a therapeutic target require further investigation.

**Methods:**

We investigated DNA methylation and demethylation changes in experimental NEC using neonatal mouse models in vivo and mouse and human enteroids in vitro. We measured global DNA methylation (5-mC), hydroxymethylation (5-hmC), and the expression of the ten-eleven translocation (TET) enzymes. We also evaluated the effects of the DNA demethylation inhibitor Bobcat339 on intestinal injury, inflammation, and the Toll-like receptor 4 (TLR4) signaling pathway.

**Results:**

Neonatal mice with NEC exhibited reduced levels of 5-hmC and increased levels of 5-mC, indicating global hypermethylation of the intestinal epithelium. Treatment with the DNA demethylation inhibitor Bobcat339 significantly reduced the severity of NEC and decreased proinflammatory mediators, including interleukin 1beta (*Il1b*), tumor necrosis factor alpha (*Tnfa*), and lipocalin-2 (*Lcn2*), following LPS-induced endotoxemia.

**Conclusions:**

These findings advance our understanding of the role of abnormal DNA demethylation in NEC development. Moreover, our results demonstrate that a DNA demethylation inhibitor attenuates experimental NEC by decreasing TLR4-driven immune responses and intestinal inflammation. Targeting DNA demethylation by modulating TLR4-associated inflammatory pathways may represent a promising therapeutic strategy for NEC.

## Introduction

Necrotizing enterocolitis (NEC) is a severe inflammatory bowel disease that predominantly affects preterm neonates[1; 2]. NEC is characterized by intestinal epithelial injury, inflammation, and necrosis, often necessitating surgical intervention. The global incidence of NEC is estimated at approximately 7% in extremely premature or very low birth weight infants (< 1,500 grams). A recent meta-analysis of over 570,000 infants revealed notable geographical variation, which has been attributed in part to differences in incidence and access to specialized care between high-income and low-income countries[3; 4]. Clinically, NEC presents with the acute onset of intestinal ischemia and necrosis and carries a mortality rate of over 20%[5]. In the most severe fulminant forms, mortality can reach up to 50% within 48 hours of diagnosis[6]. The persistently high mortality associated with NEC is largely due to the absence of disease-specific therapies[7]. Current management is primarily supportive and includes cessation of enteral feeds, gastric decompression with a nasogastric tube, hemodynamic stabilization with intravenous fluids and inotropes, and administration of broad-spectrum antibiotics targeting enteric pathogens[8]. For infants with severe disease despite medical management, surgical intervention is required, involving drainage of the abdominal cavity in the setting of intestinal perforation or resection of necrotic bowel segments[9]. Antibiotic therapy remains a cornerstone of both medical and surgical treatment for NEC, which is frequently used in the NICU setting, raising concerns about antimicrobial resistance[10]. Therefore, the development of alternative and more targeted therapeutic strategies is urgently needed to improve clinical outcomes and reduce the high mortality associated with NEC.

Among the multifactorial etiologies of NEC, excessive inflammatory signaling within the immature intestine is a central driver of tissue injury. However, the molecular mechanisms that regulate inflammatory susceptibility and disease progression in NEC remain incompletely understood. In NEC, some genes are upregulated while others are downregulated due to a complex interplay of factors, including immune response activation, epigenetic modifications, and changes in cellular processes[11; 12; 13; 14; 15; 16]. This dysregulation contributes to the development and progression of NEC. Epigenetic changes (e.g., DNA methylation, histone modifications, and non-coding RNAs) significantly influence NEC by modifying gene expression, promoting inflammation, compromising gut barrier integrity, and altering the gut microbiota balance in preterm infants[17; 18; 19; 20; 21; 22; 23; 24]. Among epigenetic markers, DNA methylation at cytosine residues in CpG dinucleotides has been widely studied for its role in transcriptional repression or activation[25]. DNA methylation dynamically changes during intestinal development and in response to environmental stimuli, influencing immune function and barrier integrity, both of which are critical in NEC pathogenesis[26; 27].

Recent high-resolution genome-wide DNA methylation profiling studies, including whole-genome bisulfite sequencing and targeted methylation assays, have revealed widespread methylation alterations in human intestinal tissue affected by NEC, with emerging evidence supporting their utility as diagnostic and prognostic biomarkers[17; 19; 20]. We previously investigated the association between DNA methylation and NEC by analyzing clinical samples from preterm infants[17; 18; 19]. Laser-captured epithelial cells from NEC surgical samples showed widespread DNA hypermethylation, less pronounced in regulatory regions (CGIs, promoters, CGI shores, and enhancers) in the ileum and colon[18]. Genome-wide methylation analysis of colon and stool samples demonstrated NEC-specific DNA hypermethylation, particularly in non-CpG island/regulatory regions. The colon-derived DNA methylation signature was also detectable in stool, providing proof of concept for stool-based NEC diagnostics[19].

Targeted bisulfite sequencing of ileal samples revealed broad hypermethylation in NEC cases, primarily outside CpG islands and promoters. Differentially methylated promoters and disrupted pathways were also identified. Importantly, stool methylation patterns mirrored tissue methylation patterns, suggesting the potential for noninvasive biomarkers for NEC[17]. A pilot study using blood samples from infants with NEC compared to age-matched controls identified distinct methylation differences during active disease[20]. Cell-type deconvolution revealed elevated signatures of ileal epithelial cells, endothelial cells, and cardiomyocytes in infants with NEC, enabling the identification of potential biomarkers via peripheral blood methylation profiling[20]. While most studies on DNA methylation in intestinal inflammation focus on hypermethylation and DNA methylation enzymes (DNMTs), recent studies suggest that demethylation enzymes, the ten-eleven translocation (TET) enzymes, TET1, TET2, and TET3, also play important roles in gut inflammation and activation of the Toll-like receptor 4 (TLR4) signaling pathway[28; 29; 30; 31; 32]. TETs catalyze DNA demethylation reactions that depend on 2-oxoglutarate and Fe(II)[33; 34; 35]. TET1 promotes M1 macrophage (pro-inflammatory) polarization via NF-κB activation and *TNFa* production, driving pro-inflammatory responses[29; 30]. TET1 inhibition attenuates inflammation, and combined glutamine-probiotic therapy downregulates TET1/DNMT1, thereby reducing iNOS demethylation and oxidative stress in intestinal barrier damage[36]. TET2 promotes hydroxymethylation of immune regulator genes, such as MyD88,[37], and is essential for epithelial repair in preclinical colitis models[38]. Although TET2 expression was upregulated during inflammation, active inflammatory bowel disease (IBD) tissues (e.g., ulcerative colitis, Crohn’s disease) often show reduced TET2 expression[28]. TET2 also modulates inflammasome activation and cytokine production[39], interacts with connexin 43 in an epigenetic feedback loop that affects inflammation and tumorigenesis[28], and is a potential therapeutic target for IBD. Furthermore, TET inhibition (e.g., IOX1) suppresses Th17-driven inflammation [40], which has previously been shown to be important in NEC pathogenesis.

In this study, we examined global DNA methylation and demethylation in human and mouse models of NEC-like intestinal injury. We also assessed how inhibiting DNA demethylation affects intestinal injury and inflammation. Since we know little about how active DNA demethylation relates to NEC development, we aimed to determine whether defects in demethylation pathways are linked to NEC and whether targeting these pathways can influence disease severity. By focusing on epigenetic regulation of inflammatory signaling, this study aims to determine the role of DNA demethylation in NEC.

## Materials and Methods

### Institutional Review Board (IRB) regulatory approvals

Intestinal tissue was obtained from an infant who required surgery for intestinal atresia. For samples acquired from infants in the NICU at Washington University in St. Louis, consent was obtained from the patients after approval by the Washington University in St. Louis School of Medicine IRB (protocol numbers #201706182 and 201804040).

#### Mouse strain and animal study approval

C57BL/6J mice were obtained from The Jackson Laboratory (Bar Harbor, ME, stock number#000664) and bred in-house. All animal procedures, including breeding, maintenance, and housing, were conducted in accordance with the *Guide for the Care and Use of Laboratory Animals* of the National Institutes of Health and were approved by the Institutional Animal Care and Use Committee (IACUC) at the University of North Carolina at Chapel Hill School of Medicine (protocol no. 24–114). All breeder mice in this study were provided ad libitum access to food and water, housed in a temperature-controlled room with a 12:12 light-dark cycle, and euthanized humanely.

#### Mouse NEC and endotoxemia models

**Figure 1:**
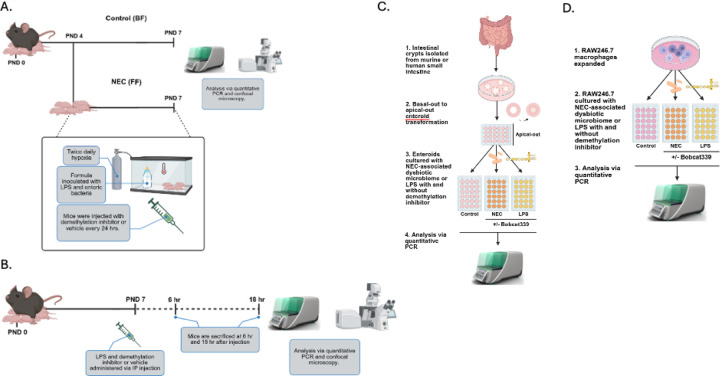
Experimental models used to investigate DNA methylation in NEC-like intestinal inflammation. **A.** Schematic of the experimental mouse NEC model. Postnatal day 4 (PND) breast milk-fed pups serve as controls (BF), while the experimental NEC formula-fed pups are exposed to NEC-associated dysbiotic bacteria, LPS, and intermittent hypoxia, and are treated with or without a demethylation inhibitor every 24 h. Ileal tissues are collected on PND7 for quantitative PCR and confocal microscopy. **B.** Schematic of the experimental mouse endotoxemia model. PND7 pups received intraperitoneal LPS (10 μg/g) with or without Bobcat339 (6.45 μg/g), and ileal tissues were harvested at 6 h and 18 h for analysis. **C.** Schematic of human and mouse enteroids NEC-in-a-dish model. Intestinal crypt-derived enteroids were converted to an apical-out configuration and exposed to NEC-associated bacteria (1 × 10^7^ CFU/mL) or LPS (100 μg/mL), with or without Bobcat339 (20 μM) for 6 h. **D.** Schematic of the RAW246.7 macrophage inflammation model. RAW246.7 cells were exposed to NEC-associated bacteria (1 × 10^4^ CFU/mL) or LPS (100 ng/mL), with or without Bobcat339 (20 μM) for 6 h. Schematics created with BioRender.com

Neonatal mouse pups were subjected to experimental NEC as previously described[41]. In brief, neonatal pups were randomly assigned to the following three experimental groups: a breast milk-fed control group (BF), a formula milk-fed with NEC-associated bacteria group (FF), and a FF group treated with a single TET inhibitor (e.g., Bobcat339 by intraperitoneal injection (IP), 6.45 μg/g body weight, once daily. The NEC-associated bacteria are dysbiotic enteric bacteria cultured from the intestine of an infant with surgical NEC. To induce experimental NEC, neonatal pups at postnatal day (PND) 4 (1.8–2.3 g body weights) were gavage-fed enteral formula with NEC-associated bacteria six times daily in 3 h increments and subjected to hypoxia (5% O_2_ and 95% N_2_) for 10 min in a chamber (Billups-Rothenberg, Inc.) twice daily for three consecutive days. Pups in the BF group remained with their dams throughout the experiment and received breast milk ad libitum. The TET inhibitor Bobcat339 (MedExpress) was administered by IP injection every 24 h from PND4 to PND6. All animals were euthanized on PND7, and ileal tissues were collected for downstream analysis (Fig. 1A).

To induce endotoxemia, PND7 neonatal pups (body weights 2.8–3.8 g) received an IP injection of lipopolysaccharide (LPS) isolated from *Escherichia coli* O127:B8 (Sigma-Aldrich) at a dose of 10 mg/kg body weight. Pups were euthanized at 6 h and 18 h following LPS injection. Ileal tissues were harvested for downstream analysis (Fig. 1B).

#### Human and mouse enteroid culture

Human enteroids and mouse enteroids were generated from neonatal ileum tissues obtained from a patient who underwent surgery for atresia or from the ileum of C57BL/6 mouse pups, respectively[42]. Briefly, freshly harvested intestinal tissue was placed in ice-cold washing medium consisting of Advanced DMEM/F12 (Thermo Fisher Scientific) supplemented with HEPES (HyClone), 10% fetal bovine serum (GeminiBio), 2 mM L-glutamine (Thermo Fisher Scientific), 100 U/mL penicillin, and 0.1 mg/mL streptomycin (Thermo Fisher Scientific). The intestinal lumen was rinsed with ice-cold Dulbecco’s phosphate-buffered saline (DPBS) (Life Technologies), opened longitudinally, and minced into small fragments. Subsequently, tissue fragments were enzymatically digested with collagenase I (Thermo Fisher Scientific) at 37°C, with continuous mechanical dissociation at 150 rpm for 20 min. The resulting suspension was then passed through a 70-μm cell strainer and washed with ice-cold washing media. After washing, the filtrate was collected on ice and centrifuged to pellet the intestinal crypts. The crypt pellet was resuspended in chilled hydrogel (Corning^®^ Matrigel^®^ Matrix, Thermo Fisher Scientific), plated, and allowed to polymerize before being overlaid with 37°C-prewarmed 50% L-WRN conditioned medium supplemented with Y-27632 (10 μM) (R&D Systems), SB-431542 (10 μM) (R&D Systems), Primocin^®^ (InvivoGen). Enteroids were maintained at 37°C in a humidified incubator containing 5% CO_2_. Media changes were performed approximately every three days, depending on enteroid confluency. Passaging was performed once a week based on growth characteristics[43]. Only enteroids that had been passaged fewer than 18 times were used.

Apical-out enteroids were generated from previously cultured basal-out enteroids using a modified version of the protocol described by Co *et al*[44]. Briefly, basal-out enteroids embedded in Matrigel were collected by scraping and resuspended in Cell Recovery Solution (Thermo Fisher Scientific) on ice for 45 min at a 1:4 (Matrigel^®^: Cell Recovery Solution) ratio to dissolve the extracellular matrix. Subsequently, the enteroids were centrifuged at 500 × *g* for 5 min at 4°C and washed twice with ice-cold DPBS. After washing, the enteroid pellet was resuspended in 37°C-prewarmed antibiotic-free 50% L-WRN conditioned medium supplemented with Y-27632 (10 μM), SB-431542 (10 μM), and plated and incubated for 48 h on a 24-well plate with ultra-low attachment, non-pyrogenic polystyrene surfaces (Millipore Sigma). For the experiments, enteroids were harvested and resuspended in antibiotic-free media containing NEC-associated bacteria (1 × 10^7^ CFU/mL) or LPS (100 μg/mL), in the presence or absence of Bobcat339 (10 μM), for 6 h (Fig. 1C).

#### Culture of mouse macrophages

RAW264.7 cells (mouse macrophage cell line; ATCC) were cultured in high-glucose DMEM containing pyruvate (Thermo Fisher Scientific) and maintained at 37°C with 5% CO_2_. For experimental treatments, cells were cultured for 24 h and then exposed to NEC-associated bacteria (1 × 10^4^ CFU/mL) or LPS (100 ng/mL), with or without Bobcat339 (10 μM), for 6 h (Fig. 1D).

### RNA isolation, cDNA preparation, and qRT-PCR

Total RNA was extracted from ileal segments collected using the RNeasy Mini Kit (QIAGEN) according to the manufacturer’s instructions. Equal amounts of total RNA were used as a template to synthesize a first-strand complementary DNA (cDNA) using the high-capacity QuantiTect^®^ Reverse Transcription Kit (QIAGEN) in a 20 μL reaction, and the volume was adjusted to 100 μL with nuclease-free water.

Quantitative real-time PCR (qRT-PCR) results were obtained using SsoAdvanced Universal SYBR Green Supermix (Bio-Rad) and specific primers on a CFX Opus Real-Time PCR Detection System (Bio-Rad). A melting curve analysis was used to determine the specificity of the amplified product. Expression levels were quantified with triplicate assays per sample, and fold change in expression was calculated using the 2^−ΔΔ^CT method. Primer-BLAST online tool and primers were synthesized by Integrated DNA Technologies, as listed in Table 1. Relative mRNA expression levels were normalized to the housekeeping gene ribosomal protein large P0 (*RPL0*) and calculated, as previously described[45; 46].

#### Immunofluorescence staining

Immunofluorescence staining was performed on 5-μm-thick paraffin-embedded tissue sections. Sections were preheated at 60°C for 30 min, deparaffinized by two washes in xylene, and sequentially rehydrated through a graded ethanol series (100%, 95%, 70%, and distilled water). Antigen retrieval was performed by immersing sections in citrate buffer (10 mM, pH 6.0) followed by microwave irradiation at 1000 W for 6 min. After washing with phosphate-buffered saline (PBS), sections were blocked with 1% bovine serum albumin (BSA) and 5% donkey serum (Sigma-Aldrich) for 1 h at room temperature. Sections were then incubated overnight at 4°C with primary antibodies diluted 1:200 in 0.5% BSA. Following three washes with PBS, appropriate fluorophore-conjugated secondary antibodies (1:1000 dilution in 0.5% BSA; Life Technologies) and the nuclear counterstain DAPI (BioLegend) were applied. Sections were mounted using ProLong^™^ Gold Antifade Reagent (Invitrogen), air-dried, and imaged using a Zeiss LSM 980 confocal microscope with appropriate filter settings[47]. The *In Situ* Cell Death Detection Kit (Fluorescein) for TUNEL staining was obtained from Roche. Terminal deoxynucleotidyl transferase dUTP nick-end labeling (TUNEL) assays were performed according to the manufacturer’s instructions to detect apoptosis. All antibodies are listed in Table 2.

### Statistical analysis

Data was analyzed using GraphPad Prism software (version 10.0.3; GraphPad Software, La Jolla, CA). Depending on the experimental design and data distribution, comparisons between groups were performed using either one-way analysis of variance (ANOVA) or an unpaired Student’s t-test, as appropriate. The Mann-Whitney test was used to perform comparisons in Supplemental Fig. 1. A *p*-value of less than 0.05 was considered statistically significant. Data are presented as mean ± standard deviation (SD), as indicated.

## Results

### Global demethylation is suppressed in an experimental mouse NEC model.

**Figure 2:**
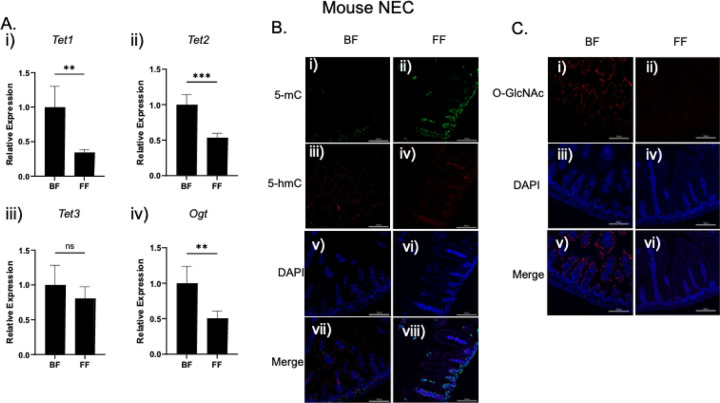
Experimental NEC is associated with suppression of DNA demethylation and disruption of the OGT-TET axis. **A.** Quantitative RT-PCR analysis of *Tets 1/2/3* (i, ii, and iii), and *Ogt* (iv) expression in mouse ileal tissue from breast milk-fed control mice (BF) and formula-fed mice with experimental NEC (FF). Each data point represents an individual mouse. **B.** Representative confocal images of ileal sections stained for 5-methylcytosine (5-mC, green, i and ii), 5-hydroxymethylcytosine (5-hmC, red, iii and iv), DAPI (blue, v and vi), and merged images (vii and viii) in BF and FF mice. FF mice demonstrated increased 5-mC staining and reduced 5-hmC staining, consistent with global DNA hypermethylation and impaired active demethylation. **C.** Representative confocal images of O-GlcNAc staining in ileal sections from BF and FF mice (red, i, and ii). DAPI (blue, iii and iv), and merged images (v and vi). Scale bars represent 100 μm. Data are shown as mean ± SD. Significance thresholds: **p < 0.01, ***p< 0.001; ns: not significant (>0.05). This experiment was performed independently three times with similar results; a representative result is shown.

To determine whether active DNA demethylation is altered in experimental NEC, we first assessed the expression of TET dioxygenases and global levels of DNA methylation and hydroxymethylation in the ileum of FF mice compared to the BF control group. qRT-PCR analysis demonstrated that expression levels of *Tet1* and *Tet2* mRNAs were significantly reduced in the FF group compared with BF controls, whereas *Tet3* mRNA expression was not significantly changed (Fig. 2A–i, -ii & -iii). In parallel, expression of O-GlcNAc transferase (*Ogt*), which functionally interacts with TET proteins to regulate chromatin-associated demethylation activity, was also significantly decreased in the FF group compared to BF controls (Fig. 2A–iv).

Consistent with reduced TET activity, immunohistochemistry revealed a marked reduction in 5-hmC levels in ileal sections from FF mice compared with BF controls. In contrast, 5-mC staining was increased in the FF group (Fig. 2B), indicating global DNA hypermethylation in experimental NEC. Furthermore, immunohistochemistry demonstrated decreased O-GlcNAcylation of serine/threonine in the intestinal epithelium of FF mice compared with BF controls (Fig. 2C), supporting disruption of the OGT-TET regulatory axis in NEC. Collectively, these findings demonstrate that experimental NEC is characterized by suppression of the active DNA demethylation pathway, reflected by decreased expression of *Tets 1*/*2*, reduced 5-hmC levels, downregulation of *Ogt*, and concomitant accumulation of 5-mC. These findings show that in experimental NEC, a disrupted OGT-TET axis inhibits active DNA demethylation, resulting in genome-wide DNA hypermethylation in the intestine.

#### DNA demethylation inhibition attenuates experimental NEC

**Figure 3:**
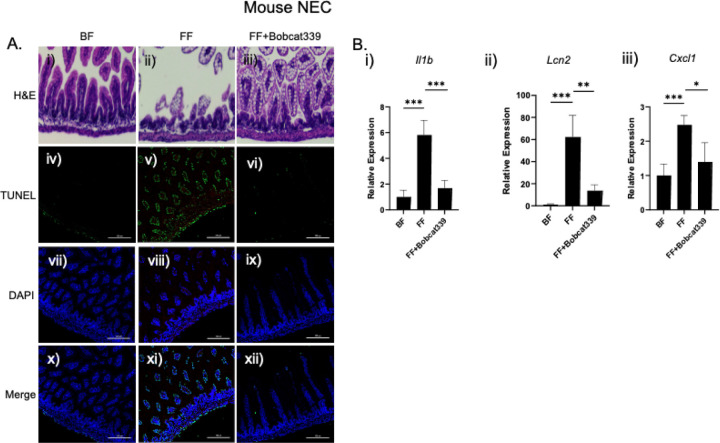
Pharmacologic inhibition of DNA demethylation attenuates intestinal injury, epithelial apoptosis, and inflammation in experimental NEC. **A.** Representative ileal sections from BF control mice, mice subjected to experimental NEC (FF), and FF mice treated with the DNA demethylation inhibitor Bobcat339. Hematoxylin and eosin (H&E, i, ii, and iii) staining demonstrates mucosal injury and villus disruption in FF mice, with preservation of the intestinal architecture following Bobcat339 treatment. Representative confocal images with TUNEL staining (iv, v, and vi) identified increased apoptotic cells (green) in FF mice, which were reduced with Bobcat339 treatment. DAPI nuclear counterstaining (blue, vii, viii, and ix) with merged images (x, xi, and xii). Scale bars represent 100 μm. Data are shown as mean ± SD. **B.** Quantitative RT-PCR analysis of ileal proinflammatory marker expression *Il1a* (i), *Lcn2* (ii), *Cxcl1* (iii). Treatment with Bobcat339 significantly reduced NEC-associated induction of proinflammatory mediators. Each data point represents an individual mouse. Data are shown as mean ± SD. Significance thresholds: *p < 0.05: **p < 0.01, ***p < 0.001; ns: not significant (>0.05).

We next investigated whether pharmacologic inhibition of DNA demethylation alters NEC severity. Neonatal mice were subjected to our established NEC protocol[41]. FF mice exhibited overt intestinal injury, including ischemic discoloration, as well as significant mucosal destruction and inflammatory cell infiltration on H&E staining compared to BF controls (Fig. 3A–i, -ii & -iii). In contrast, FF mice treated daily with the TET inhibitor Bobcat339 (FF+Bobcat339) showed markedly improved gross morphology and preservation of villus architecture. FF mice with NEC demonstrated abundant epithelial apoptosis, evidenced by increased TUNEL staining (Fig. 3A–iv, -v & -vi). These pathological features were substantially attenuated by Bobcat339 treatment, approaching levels observed in BF controls. At the molecular level, FF mice with NEC exhibited significantly elevated expression of *Il1b*, *Lcn2*, and *Cxcl1*, while Bobcat339 administration markedly suppressed *Il1b*, *Lcn2*, and *Cxcl1* expression in the ileum, indicating reduced inflammation (Fig. 3B). Together, these data demonstrate that inhibition of DNA demethylation significantly mitigates intestinal injury, apoptosis, and inflammatory signaling in experimental NEC.

### DNA demethylation inhibitors attenuate gut injury through the TLR4 signaling pathway in the experimental mouse NEC model.

**Figure 4:**
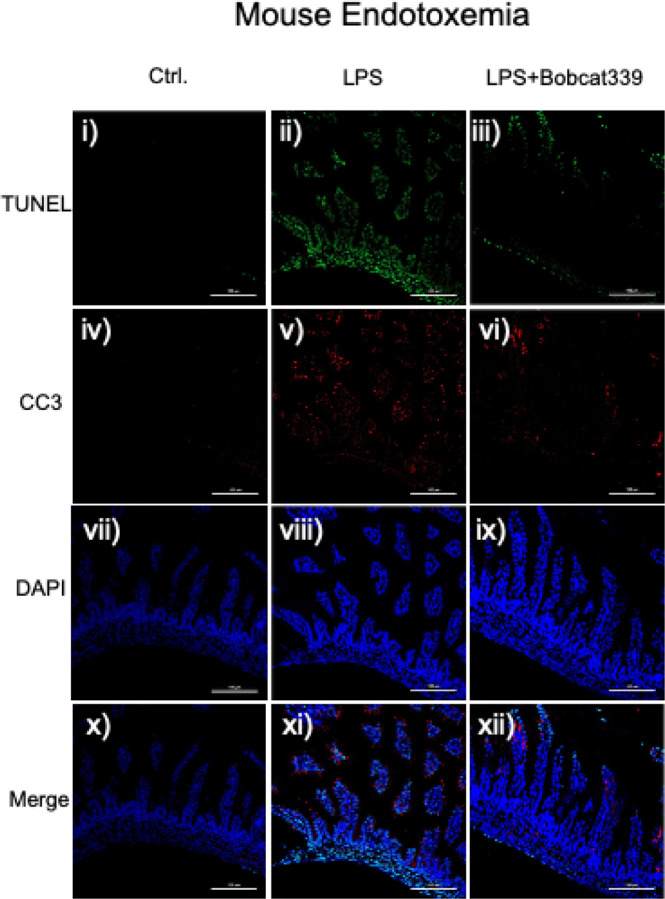
DNA demethylation inhibition suppresses LPS-induced apoptosis and TLR4-associated inflammatory signaling in neonatal mice. - Representative confocal images of ileal sections from control (Ctrl) mice, LPS-treated mice, and LPS-treated mice receiving Bobcat339. Sections were stained for TUNEL (green, i-iii), cleaved caspase 3 (CC3, red, an apoptosis marker, iv-vi), counterstained with DAPI (nuclear, blue, vii-ix), and merged images (x-xii). LPS administration increased intestinal epithelial apoptosis, while Bobcat339 reduced TUNEL and CC3 staining. Scale bars represent 100 μm.

Since the LPS-TLR4-NFκB signaling pathway is central to NEC pathogenesis[48], we next examined whether inhibition of DNA demethylation modulates TLR4-dependent inflammatory responses using an LPS-induced neonatal endotoxemia model. Neonatal mice subjected to LPS-induced endotoxemia demonstrated abundant epithelial apoptosis, evidenced by increased TUNEL and cleaved caspase-3 staining (Fig. 4). These pathological features were substantially attenuated by Bobcat339 treatment, approaching levels observed in controls. Treatment with Bobcat339 significantly reduced inflammation and apoptosis in this endotoxemia model. Notably, LPS exposure also decreased the expression of *Tets 1/2/3* (**Suppl. Figure 1**), consistent with observations in the NEC model. These findings indicate that inhibition of DNA demethylation suppresses TLR4-mediated inflammatory activation in vivo.

### DNA demethylation inhibition suppresses inflammatory activation in a murine NEC-in-a-dish model.

**Figure 5:**
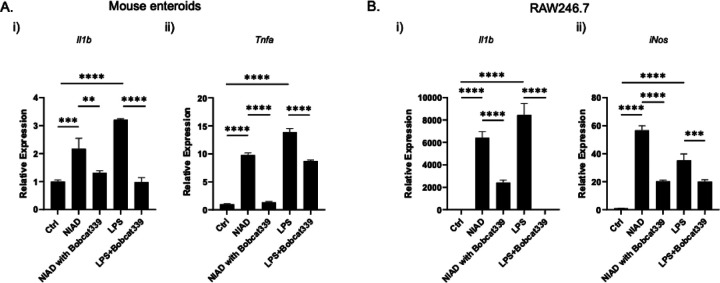
Inhibition of DNA demethylation suppresses inflammatory activation in mouse enteroid and macrophage NEC-in-a-dish models. **A.** Mouse enteroid NEC-in-a-dish (NIAD) model: Mouse enteroids were exposed to NEC-associated enteric bacteria (1 × 10^7^ CFU/mL) from a patient with NEC or LPS (100 μg/ml) in the presence or absence of Bobcat339 (20 μM) for 6 h. Quantitative RT-PCR analysis demonstrated induction of *Il1b* (i) and *Tnfa* (ii) in the NIAD or LPS models, which were significantly reduced by Bobcat339 treatment. **B.** RAW246.7 macrophages were exposed to NEC-associated bacteria (1 × 10^4^ CFU/mL) or LPS (100 ng/mL) with or without Bobcat339 (20 μM) for 6 h. Quantitative RT-PCR analysis demonstrated induction of *Il1b* (i) and *iNOS* (ii) in the NIAD and LPS-exposed groups, which was significantly attenuated by Bobcat339. Data are shown as mean ± SD. Significance thresholds: **p < 0.01, ***p < 0.001, ****p < 0.0001.

To determine whether the protective effects of demethylation inhibition are epithelial- and macrophage-intrinsic, we utilized mouse enteroid and RAW264.7 macrophage models. Exposure of mouse enteroids to NEC-associated bacteria (NEC-in-a-dish) or LPS significantly induced *Il1b* and *Tnfa* expression (Fig. 5A), where treatment with Bobcat339 markedly suppressed this cytokine induction. Similarly, in RAW264.7 macrophages, NEC-associated bacteria or LPS increased *Il1b* and *iNos* expression (Fig. 5B), and Bobcat339 treatment significantly attenuated this response. These data demonstrate that inhibition of DNA demethylation directly dampens inflammatory activation in both intestinal epithelial and immune cells.

### DNA demethylation inhibition suppresses cytokine induction in human enteroid NEC models.

To translate these findings to the human condition, we utilized a model of human neonatal intestinal enteroids.

**Figure 6:**
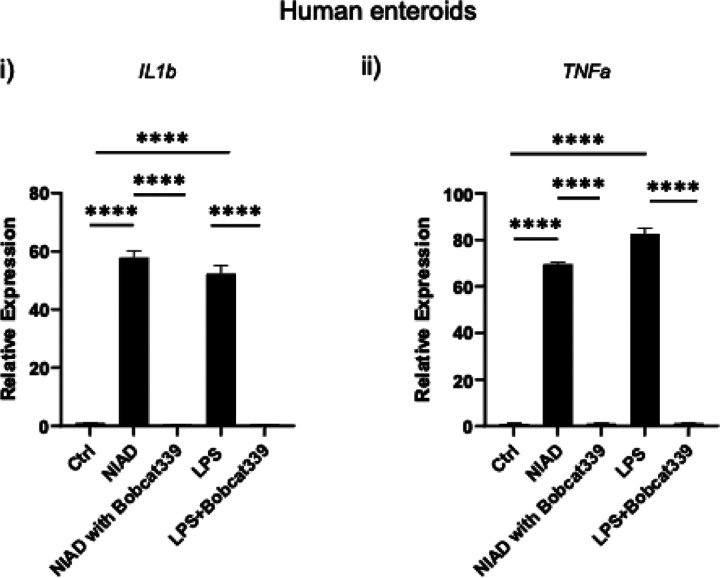
Inhibition of DNA demethylation suppresses cytokine induction in human enteroid NEC-in-a-dish models. **A.** Human neonatal enteroids were exposed to NEC-associated enteric bacteria (1 × 10^7^ CFU/mL) or LPS (100 μg/mL) in the presence or absence of the DNA demethylation inhibitor Bobcat339 (20 μM) for 6 h. Quantitative RT-PCR analysis demonstrated increased expression of *IL1b* (i) and *TNFa* (ii) following NIAD or LPS exposure. Bobcat339 treatment significantly reduced the cytokine induction in both experimental conditions. Data are shown as mean ± SD. Significance thresholds: ****p < 0.0001.

Human neonatal intestinal enteroids cultured with NEC-associated bacteria showed significant induction of *IL1b* and *TNFa* expression, and LPS similarly triggered cytokine upregulation (Fig. 6). In both in vitro NEC conditions, Bobcat339 treatment significantly reduced inflammatory gene expression. These findings show that inhibition of DNA demethylation suppresses inflammatory responses in the human intestinal epithelium, thereby supporting translational relevance.

## Discussion

In this study, we demonstrate that experimental NEC is characterized by global DNA hypermethylation, reduced expression of TET dioxygenases, diminished 5-hmC, and heightened TLR4-driven inflammatory signaling. Pharmacologic inhibition of DNA demethylation significantly attenuated intestinal injury in vivo, inflammatory mediators, and suppressed proinflammatory cytokine induction in murine and human in vitro NEC models. Collectively, these findings identify dysregulated DNA demethylation as a novel contributor to NEC pathogenesis and suggest that targeting epigenetic demethylation pathways may represent a new potential therapeutic strategy.

NEC is characterized by intestinal ischemia and hypoxia resulting from impaired perfusion and inflammatory injury. Hypoxic stress disrupts epithelial barrier integrity and alters cellular metabolism and microbial composition, favoring anaerobic organisms such as *Clostridium* species[2]. Importantly, TET enzymes require oxygen and α-ketoglutarate to catalyze oxidative DNA demethylation[49]. Thus, hypoxia may directly suppress TET activity, shifting the epigenetic balance toward DNA hypermethylation. Our observation of reduced *Tet1* and *Tet2* expression and diminished 5-hmC levels in experimental NEC is consistent with prior reports of global DNA hypermethylation in human NEC tissue, stool, and blood samples[17; 18; 19; 20]. However, global hypermethylation should not be equated with uniform transcriptional repression. The inflamed intestine is composed of heterogeneous epithelial, stromal, and immune populations, each contributing distinct methylation signatures. Moreover, NEC-associated methylation changes occur predominantly outside promoters and CpG islands[17], suggesting remodeling of regulatory elements rather than widespread gene silencing. These data support a model in which NEC pathogenesis is driven by gene- and cell-type-specific epigenetic reprogramming rather than global transcriptional repression.

A central finding of this study is that inhibition of DNA demethylation attenuates intestinal inflammation, at least in part, by suppressing TLR4-mediated inflammatory signaling. The DNA demethylation inhibitor Bobcat339 reduced inflammatory mediators in both NEC and endotoxemia models. These data suggest that active demethylation may enhance inflammatory responsiveness by promoting accessibility of TLR4 regulatory regions. Mechanistically, we propose that TET-dependent demethylation at TLR4 promoters or enhancers may facilitate transcriptional activation in response to microbial or hypoxic stimuli. Inhibition of DNA demethylation may therefore limit chromatin accessibility and dampen downstream NF-κB-mediated cytokine cascades. Although locus-specific methylation analysis was not performed in this study, our functional data strongly support epigenetic regulation upstream of TLR4-dependent inflammation.

Interestingly, acute inflammatory stimuli can induce TET expression as part of immediate-early transcriptional responses, whereas sustained inflammation, oxidative stress, or metabolic exhaustion may ultimately suppress TET expression. Thus, DNA demethylation may contribute to early disease amplification even if global TET levels decline during later stages of injury. Extensive prior work demonstrates that TET enzymes exert highly context-dependent effects on immune regulation. In macrophages, TET2 has been shown to restrain inflammatory gene expression in certain settings (e.g., LPS treatment and during *E. coli* infection)[50; 51], yet promotes locus-specific demethylation of TAB2, a key molecule of the TLR4/MAPK signaling pathway [32]. Consistent with our findings, a previous study demonstrated that TET1 promotes M1 macrophage polarization (proinflammatory) by activating the NF-κB signaling pathway [29]. In THP-1 cells, TET1 knockdown significantly reduced the expression of M1-associated inflammatory mediators, including IL6, TNFa, CCL2, and HLA-DR, in response to Porphyromonas gingivalis LPS/IFN-γ stimulation, and these effects were mediated through suppression of NF-κB signaling[29]. Consistent with our data (Fig. 5B), treatment with a TET inhibitor significantly reduced the upregulation of *Il1b* and *iNos* in RAW264.7 macrophages exposed to NEC-associated dysbiotic bacteria or LPS. Together, these findings suggest that TET-mediated DNA demethylation facilitates NF-κB-dependent inflammatory activation and M1 macrophage polarization and that inhibition of this pathway dampens macrophage proinflammatory responses. Ansari *et al*. reported that conditional deletion of *Tet2 and Tet3* in mouse intestinal epithelial cells disrupts cellular composition, leading to a marked loss of Paneth cells, reduced Tuft cells, and an increase in enteroendocrine cells, indicating impaired secretory lineage differentiation[38]. Additionally, loss of TETs 2/3 alters gut microbiota composition, promoting a pro-inflammatory state under basal conditions and increasing susceptibility to inflammation-induced mortality in mice[38]. On the other hand, TETs 2/3 are required for regulatory T cell stability, and their loss promotes Th17-mediated inflammation[52; 53]. These findings highlight that TET activity must be tightly regulated, particularly in developmentally immature tissues such as the neonatal intestine. Beyond adaptive immunity, TET enzymes regulate iNKT cell lineage specification. Combined deletion of TET2 and TET3 skews thymocytes toward an NKT17 phenotype characterized by elevated IL-17 production and reduced T-bet and ThPOK expression[54].

These findings underscore the need to tightly regulate TET activity during immune development and inflammatory responses, particularly in the developmentally immature neonatal intestine. *Tet2*-deficient myeloid cells further illustrate this complexity. Loss of *Tet2* can amplify *Il6* and *Il1b* gene expression and promote inflammasome activation[50; 55], contributing to systemic inflammatory disorders, including cardiovascular disease and clonal hematopoiesis–associated pathologies[56; 57; 58; 59]. In hematopoietic stem cells, the inflammatory phenotype is more nuanced. *TET2*-mutant hematopoietic stem cells can create a proinflammatory milieu via myeloid progeny[60]. External inflammatory signals, like *IL1b* or *IL6*, preferentially expand *Tet2*-deficient clones, forming a feed-forward loop where inflammation promotes clone expansion, and mutant cells amplify inflammation[61; 62]. Together, these data highlight that TET enzymes can either restrain or amplify inflammation depending on cellular context, developmental stage, and metabolic state.

Our findings place NEC within this framework of context-dependent epigenetic regulation. Bobcat339 is a widely used chemical that has advanced the understanding of TET-dependent DNA demethylation in inflammatory regulation, originally developed as a cytosine-based TET inhibitor[63], with the chemical name 1-([1,1′-Biphenyl]-3-yl)-4-amino-5-chloropyrimidin-2(1H)-one. It has been applied in models of renal ischemia, cancer, and inflammatory disease, where it modulates *IL1b-*driven transcriptional programs[63; 64]. Inhibition of TET2 can also exacerbate NLRP3 inflammasome activation and vascular calcification in other settings[65]. Bobcat339 has also served as a mechanistic tool to confirm the TET dependence of vitamin C-mediated epigenetic modulation and ferroptosis-related pathways[66; 67].

In our NEC models, Bobcat339 consistently attenuated inflammatory signaling and tissue injury in vivo and in vitro. Importantly, these findings do not imply that global TET inhibition is universally protective. Rather, they suggest that in the specific context of neonatal intestinal inflammation, active DNA demethylation contributes to amplification of TLR4-mediated injury. The divergent effects of TET inhibition across TET inhibitor doses highlight isoform-, cell type-, and disease-specific roles of DNA demethylation.

We also observed reduced *Ogt* expression in experimental NEC. OGT forms a chromatin-associated complex with TET proteins, modulating DNA demethylation and histone modifications in response to the cellular metabolic state[68; 69; 70; 71]. Disruption of this axis has been shown to alter global and locus-specific methylation patterns[72]. Given the metabolic stress seen in NEC, impaired OGT-TET coupling may further dysregulate epigenetic control of inflammatory gene expression, linking nutrient availability to inflammatory susceptibility in the premature intestine.

NEC primarily affects preterm infants, whose intestines are developmentally immature at both structural and epigenetic levels. Premature birth is associated with dynamic and incompletely stabilized DNA methylation landscapes. Moreover, TLR4 expression is physiologically elevated in the developing intestine and contributes to normal maturation[73]. TET enzymes are essential for intestinal stem cell maintenance, epithelial differentiation, and immune homeostasis[74; 75]. We propose that aberrant TET-mediated demethylation of TLR4 regulatory elements in the premature intestine may amplify inflammatory responsiveness to microbial colonization or hypoxic stress, thereby predisposing to NEC. Future studies examining locus-specific methylation at TLR4 and related inflammatory genes in preterm infants may identify novel biomarkers and therapeutic targets.

Several limitations in this study warrant consideration. We did not perform genome-wide or locus-specific methylation mapping to directly demonstrate TLR4 promoter demethylation. Cell type-specific contributions of epithelial versus immune TET activity remain to be defined. Additionally, long-term safety and developmental effects of TET inhibition in neonates require careful evaluation. Future studies incorporating chromatin accessibility assays, cell-type-specific conditional knockouts, and analyses of human preterm intestine with NEC will be critical to delineate the precise epigenetic circuitry involved.

## Conclusion

In summary, our data demonstrate that experimental NEC is associated with suppressed TET expression, reduced 5-hmC formation, global DNA hypermethylation, and exaggerated TLR4-mediated inflammation. We also show that pharmacologic inhibition of DNA demethylation attenuates intestinal injury across multiple experimental platforms. These findings identify DNA demethylation as a mechanistic regulator of inflammatory susceptibility in NEC-mediated inflammation and suggest that selective modulation of epigenetic pathways may offer a novel therapeutic avenue for this devastating neonatal disease.

## Supplementary Material

Supplementary Files

This is a list of supplementary files associated with this preprint. Click to download.

- Tables.docx
- SupplementalFiglegend.docx

## Abbreviations

5-hmC: 5-hydroxymethylcytosine
5-mC: 5-methylcytosine
ANOVA: analysis of variance
BF: breast milk-fed controls
BLAST: Basic Local Alignment Search Tool
BMDM: bone marrow-derived macrophages
BSA: bovine serum albumin
CC3: cleaved caspase-3
CCL2: C-C motif chemokine ligand 2
CD4: cluster of differentiation 4
CD8: cluster of differentiation 8
cDNA: complementary DNA
CFU: colony-forming units
CGI: CpG island
CpG: 5’-cytosine nucleotide-phosphate-guanine nucleotide-3’
Ctrl: control
CXCL-1: C-X-C motif chemokine ligand 1
CXCL-2: -X-C motif chemokine ligand 2
DAPI: 4’,6-diamidino-2-phenylindole
DMEM: Dulbecco’s Modified Eagle Medium_
DMEM/F12: Dulbecco’s Modified Eagle Medium/Ham’s F-12 Nutrient Mixture
DNA: deoxyribonucleic acid
DNMT: DNA methyltransferase
DNMT1: DNA methyltransferase 1
DPBS: Dulbecco’s phosphate-buffered saline
FF: formula milk-fed with enteric bacteria from a patient with NEC
FoxP3: forkhead box P3
HDAC: histone deacetylase
H&E: hematoxylin and eosin
HEPES: 4-(2-hydroxyethyl)piperazine-1-ethanesulfonic acid
IACUC: Institutional Animal Care and Use Committee
IFN-g: interferon gamma
IgG: immunoglobulin G
IL17: interleukin-17
IL1B and Il1b: interleukin1beta gene
IL6: interleukin 6 gene
iNKT: invariant natural killer cells
iNOS: inducible nitric oxide synthase
IOX1: 8-hydroxyquinoline-5-carboxylic acid
IP: intraperitoneal injection
IRB: Institutional Review Board
LCN2: lipocalin-2
LPS: lipopolysaccharide
L-WRN: Wnt-3A, R-spondin 3, and noggin conditioned medium from an L cell line
MAPK: mitogen-activated protein kinase
MyD88: myeloid differentiation primary response 88
NCBI: National Center for Biotechnology Information
NEC: necrotizing enterocolitis
NF-κB: nuclear factor kappa-light-chain-enhancer of activated B cells
NIAD: NEC in a dish
NICU: neonatal intensive care unit
NIH: National Institutes of Health
NKT1: natural killer T-1 cells
NKT17: natural killer T-17 cells
NKT2: natural killer T-2 cells
O-GlcNAc: O-linked β-*N*-acetylglucosamine
OGT: O-GlcNAc transferase
PBS: phosphate-buffered saline
PND: postnatal day
RNA: ribonucleic acid
RPL0: ribosomal protein large P0
rpm: revolutions per minute
qRT-PCR: quantitative real-time polymerase chain reaction
TAB2: TAK1 binding protein 2
T-bet: T-box expressed in T cells
TET1: ten-eleven translocation enzyme 1
TET2: ten-eleven translocation enzyme 2
TET3: ten-eleven translocation enzyme 3
Tfh: T follicular helper
Th17 cells: T helper 17 cells
THP-1: Tohoku Hospital Pediatrics-1
ThPOK: T-helper inducing POZ/Krüppel-like factor
TLR4: toll-like receptor 4
TNFa: tumor necrosis factor alpha
Treg cells: regulatory T cells
TUNEL: terminal deoxynucleotidyl transferase dUTP nick end labeling
